# Therapeutic Opportunities in Overcoming Premature Termination Codons in Epidermolysis Bullosa via Translational Readthrough

**DOI:** 10.3390/cells14151215

**Published:** 2025-08-07

**Authors:** Kathleen L. Miao, Ryan Huynh, David Woodley, Mei Chen

**Affiliations:** Department of Dermatology, The Keck School of Medicine, University of Southern California, Los Angeles, CA 90033, USA

**Keywords:** aminoglycosides, epidermolysis bullosa, gentamicin, nonsense suppression therapy, premature termination codon, readthrough therapy

## Abstract

Epidermolysis Bullosa (EB) comprises a group of inherited blistering disorders caused by pathogenic variants in genes essential for skin and mucosal integrity. Nonsense mutations, which generate premature termination codons (PTCs), result in reduced or absent protein expression and contribute to severe disease phenotypes in EB. Readthrough therapies, which may continue translation past PTCs to restore full-length functional proteins, have emerged as promising approaches. This review summarizes findings from preclinical studies investigating readthrough therapies in EB models, clinical studies demonstrating efficacy in EB patients, and emerging readthrough agents with potential application to EB. Preclinical and clinical studies with gentamicin have demonstrated restored type VII collagen and laminin-332 expression, leading to measurable clinical improvements. Parallel development of novel compounds—including aminoglycoside analogs (e.g., ELX-02), translation termination factor degraders (e.g., CC-90009, SRI-41315, SJ6986), tRNA post-transcriptional inhibitors (e.g., 2,6-diaminopurine, NV848), and nucleoside analogs (e.g., clitocine)—has expanded the therapeutic pipeline. Although challenges remain regarding toxicity, codon specificity, and variable protein restoration thresholds, continued advances in molecular targeting and combination therapies offer the potential to establish readthrough therapies as localized or systemic treatments addressing both cutaneous and extracutaneous disease manifestations in EB.

## 1. Epidermolysis Bullosa

Epidermolysis Bullosa (EB) encompasses a group of inherited cutaneous conditions hallmarked by blistering and scarring of the skin and mucosal surfaces [1]. To date, pathogenic variants have been identified in 16 genes that encode proteins essential for preserving the structural and functional integrity of the dermal–epidermal junction (DEJ). Genetic alterations that lead to decreased or absent protein function compromise not only tissue stability but also key cellular processes vital to tissue repair and barrier maintenance [2]. The resulting genetic heterogeneity contributes to significant variability in clinical severity, ranging from mild, localized blistering to more extensive erosions that can be accompanied by complications. Moreover, disease-associated genes may be widely expressed in other epithelial tissues (gastrointestinal, respiratory, and urogenital tracts) or in mesenchymal organs (smooth and skeletal muscle), thus rendering the more severe EB variants as systemic disorders marked by multi-organ involvement, secondary extracutaneous manifestations, and heightened morbidity and mortality [3].

Hereditary EB is classified into four major types based on the level of cleavage within the skin [2,4]. EB simplex exhibits blistering confined to the epidermis, predominantly caused by mutations in genes encoding keratins 5 and 14 or plectin (*KRT5*, *KRT14*, *PLEC*). Junctional EB (JEB) results from cleavage within the lamina lucida due to mutations in genes encoding laminin 332 (*LAMA3*, *LAMB3*, *LAMC2*), type XVII collagen (*COL17A1*), integrin α6β4 *(ITGA6*, *ITGB4*), or the integrin α3 subunit (*ITGA3*). In dystrophic EB (DEB), blistering occurs below the lamina densa, arising from defects in genes encoding type VII collagen (*COL7A1*). Kindler EB, which involves variable levels of tissue separation, is linked to mutations in *KIND1* (also known as *FERMT1*) [5]. These categories are further distinguished by inheritance pattern, genotype, phenotype, and clinical and molecular features [6].

EB remains incurable, and current treatments center on meticulous wound care, prevention of secondary complications, and management of symptoms such as pruritus and pain, along with measures to prevent infection, optimize nutrition, and provide psychosocial and occupational therapy [7,8,9,10]. Investigational therapies include gene, cell, and protein therapy, interventions that target secondary disease mechanisms such as fibrosis, and approaches based on stop codon readthrough [11,12]. To date, these treatment opportunities remain mostly experimental or are in early clinical stages. However, three notable developments recently approved by the FDA include Vyjuvek™, a herpes simplex virus 1 vector–based gene therapy; Filsuvez^®^, a birch bark extract; and Zevaskyn™, an autologous cell sheet-based gene therapy [13,14,15].

## 2. Introduction to Readthrough Therapies

Nonsense mutations that generate premature termination codons (PTCs) tend to result in the complete loss of full-length protein expression, resulting in detrimental phenotypic effects. In recessive DEB (RDEB), such nonsense mutations occur in approximately 10–25% of patients, whereas in JEB about 95% of *LAMB3* mutations—accounting for over 80% of severe JEB cases—are of this type [16,17]. When a ribosome encounters a PTC, translation prematurely terminates, producing a truncated, nonfunctional protein. To mitigate potential harm to cellular homeostasis, nonsense-mediated decay (NMD) serves as a protective mechanism by selectively degrading mRNA transcripts containing PTCs [18,19].

A primary strategy to treat nonsense mutations involves promoting ribosomal readthrough, in which an amino acid is inserted at the PTC site, effectively allowing the ribosome to continue translation. This process prevents both premature termination and NMD, encouraging the synthesis of a functional, full-length protein. Multiple factors dictate the success of readthrough therapies, including (1) mRNA transcript levels—shaped by gene expression and NMD activity, (2) the identity, location, and sequence context of the PTC, and (3) the properties of the affected protein [20].

The concept of readthrough therapy for genetic diseases was first demonstrated in 1996 by Howard et al., who showed that aminoglycosides could induce readthrough and restore functional cystic fibrosis transmembrane conductance regulator (CFTR) protein in cystic fibrosis (CF) by targeting the p.Gly542* mutation [21]. Treatment with 0.1 mg/mL G418 resulted in up to 35% of the wild-type CFTR protein level in vitro. Since then, numerous reports have confirmed the value of readthrough therapies for a range of diseases linked to nonsense mutations [22]. Although some compounds act directly on the ribosomal decoding center, others can affect distinct steps in the translation process [23].

**Figure 1:**
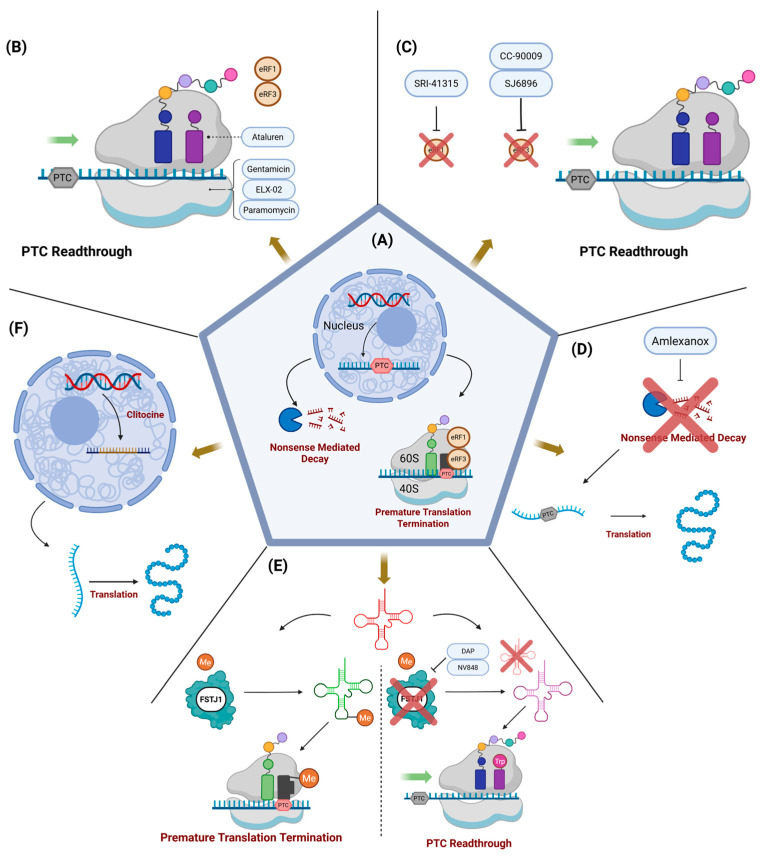
Mechanisms of Readthrough and Nonsense Suppression Strategies. (**A**) Under normal conditions, transcripts with premature termination codons (PTCs) are degraded via nonsense-mediated decay (NMD) or translated into truncated peptides due to release factor-mediated termination. (**B**) Aminoglycosides (gentamicin, paromomycin), ELX-02, and ataluren promote PTC readthrough by binding the ribosomal A site—either facilitating near-cognate tRNA incorporation or inhibiting release factor activity. (**C**) Translation termination factor inhibitors (CC-90009, SJ6896, SRI-41315) reduce levels of eRF3a or eRF1, impairing termination and enabling readthrough. (**D**) Amlexanox inhibits NMD, increasing PTC-containing mRNA available for basal readthrough. (**E**) DAP and NV848 inhibit FtsJ RNA 2’-O-methyltransferase (FTSJ1), an enzyme involved in tRNA modifications required for accurate UGA recognition, promoting readthrough. (**F**) Clitocine substitutes for adenosine in mRNA, disrupting stop codon formation and preventing termination. Illustration created with BioRender©, accessed on 2 August 2025.

**Figure 2:**
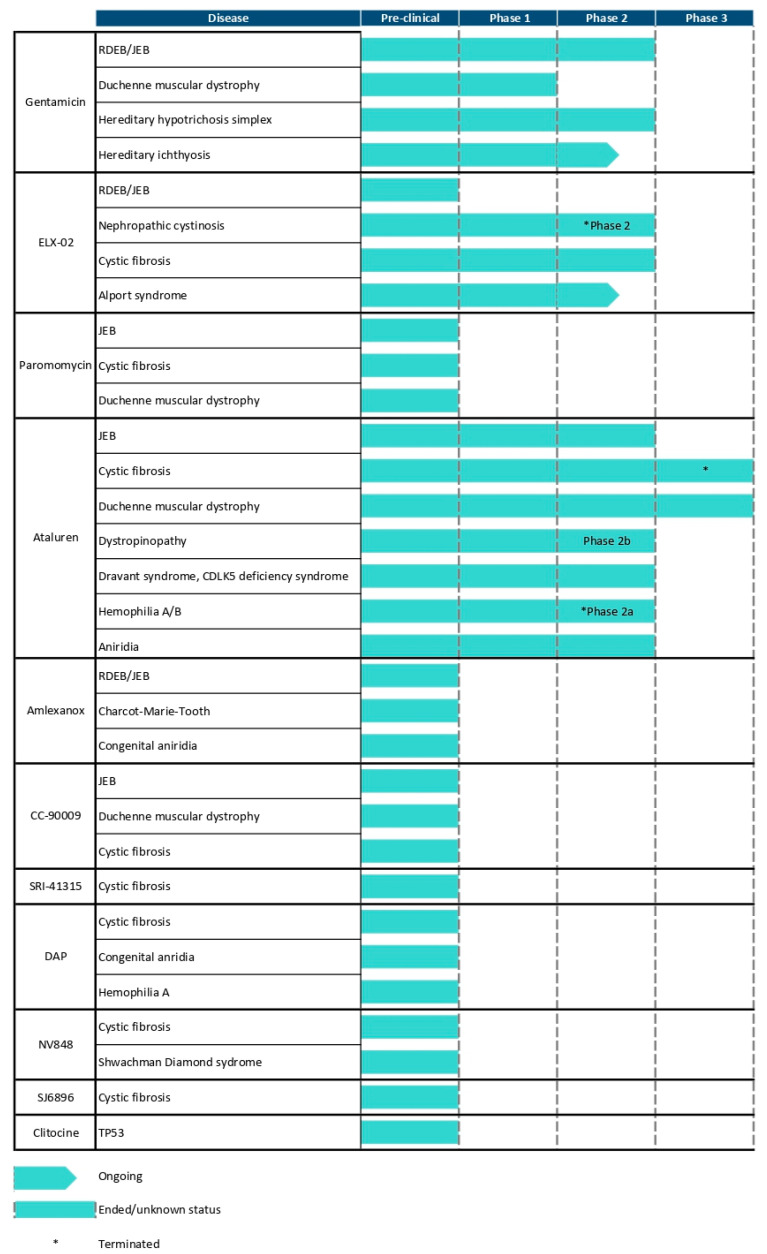
Clinical Trial Status of Aforementioned Readthrough Therapies. Overview of clinical trial progress for readthrough compounds from February 2000 to May 2025. The development of ataluren for nonsense mutation-induced CF was discontinued following negative results from a phase 3 trial. In certain instances, compounds had already received FDA approval for other indications, and drug repurposing has facilitated expedited progression through the clinical trial pipeline.

In this review, we summarize current findings on (1) established readthrough therapies for EB that have progressed through clinical evaluation; and (2) readthrough therapies tested exclusively in preclinical EB studies in in vitro models. We also examine emerging readthrough candidates that have shown promise in non-EB disorders, discussing their potential to inform future therapeutic directions for EB. Figure 1 summarizes the proposed mechanisms of action of current and potential readthrough therapies for EB, while Figure 2 details the clinical trial status of these readthrough therapies.

## 3. Aminoglycoside-Mediated Readthrough for EB

### 3.1. Introduction to Aminoglycosides

Aminoglycosides are a class of antibiotics with the unique ability to bind to the decoding center at the A site of the 40S ribosomal subunit, leading to the incorporation of near-cognate tRNAs that are able to continue readthrough past PTCs [24]. Aminoglycosides were among the first compounds identified to promote PTC readthrough and remain the most extensively studied in this context, showing potential to restore protein function in diseases such as certain cancers, neuromuscular disorders, and neurodegenerative conditions [24]. Paromomycin was among the first aminoglycosides to report nonsense mutation suppression in mammalian cells in 1985. Since then, it has been well characterized as a readthrough therapy, though strictly in preclinical studies for CF, Duchenne Muscular Dystrophy (DMD), RDEB, and JEB [25,26,27,28,29]. Meanwhile, gentamicin has emerged as the most well-characterized readthrough agent among aminoglycosides, with demonstrated efficacy in numerous in vitro and in vivo models, and has advanced into early-phase clinical trials.

One of the earliest demonstrations of gentamicin’s readthrough capacity occurred in 1996, when it was shown to restore CFTR protein expression in HeLa cells transfected with CFTR nonsense mutations (p.Gly542* and p.Arg553*) associated with CF [21]. Subsequent studies extended these findings to genetic disorders like Ataxia–Telangiectasia, where gentamicin treatment of patient-derived lymphoblastoid cells led to the production of functional Ataxia Telangiectasia Mutated (ATM) protein [30]. In the mouse model for DMD, gentamicin restored dystrophin expression to 10–15% of wild-type levels [31]. Early clinical studies in CF patients with PTCs revealed promising outcomes, with gentamicin inducing full-length CFTR protein expression in a subset of participants [32,33,34]. However, outcomes have varied across diseases; in one study, gentamicin treatment in patients with DMD and Becker Muscular Dystrophy did not lead to detectable dystrophin re-expression, highlighting the context-dependent efficacy of aminoglycoside-based therapies [35,36].

**Figure 3:**
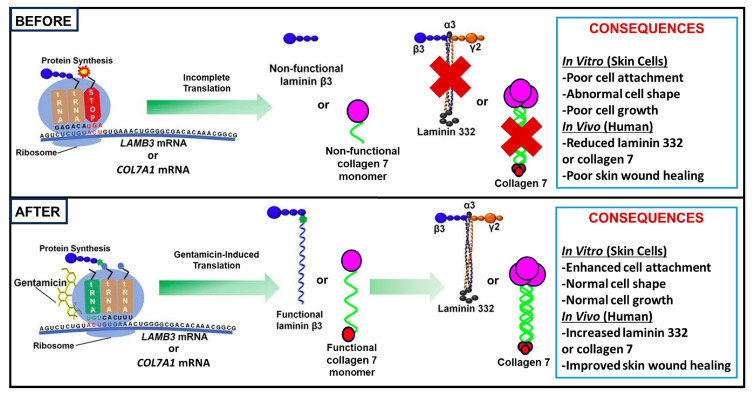
Gentamicin-induced readthrough in RDEB and JEB. In the top panel, a nonsense mutation causes premature translation termination, leading to a truncated, non-functional protein, such as C7 for RDEB or laminin β3 for JEB. The bottom panel demonstrates how gentamicin enables translation to proceed beyond the mutation, resulting in the production of a full-length, functional protein. This process corrects RDEB and JEB subtypes in vitro, increases levels of laminin 332 and C7, and enhances wound healing in human patients.

In the context of EB, our laboratory has conducted several preclinical and clinical studies demonstrating aminoglycoside-mediated readthrough for RDEB and JEB, with successful restoration of type VII collagen (C7) and laminin 332, respectively. Our work has mostly focused on gentamicin specifically, with the mechanism of gentamicin-induced PTC readthrough in RDEB and JEB illustrated in Figure 3. In the following sections, we will further elaborate on our results, as well as subsequent similar studies, using aminoglycosides as a successful therapy for EB.

### 3.2. Studies on RDEB

In preclinical studies of RDEB, gentamicin and paromomycin have both shown promising results in promoting readthrough of *COL7A1* nonsense mutations. In our study, gentamicin and paromomycin induced dose-dependent induction of C7 in RDEB fibroblasts and keratinocytes harboring *COL7A1* nonsense mutations [27]. Furthermore, gentamicin treatment of patient-derived cells harboring PTCs in the *COL7A1* gene led to re-expression of C7 at levels sufficient to reverse the abnormal cellular phenotype and enable proper incorporation of C7 into the DEJ in cultured skin equivalents [27]. Further validation in cells transfected with 22 different *COL7A1* nonsense mutations demonstrated the broader applicability of aminoglycoside-mediated readthrough in RDEB [27]. This study supported gentamicin’s therapeutic potential for restoring essential protein function in RDEB caused by nonsense mutations. From here, multiple clinical studies have since explored different dosing regimens and formulations of gentamicin to optimize its safety and efficacy in RDEB patients.

For clinical trials studying RDEB specifically, topical gentamicin, intradermal gentamicin, and intravenous (IV) gentamicin have all been explored as potential delivery methods for promoting readthrough of nonsense mutations. Our study group conducted a phase 1/2 clinical trial evaluating the safety and efficacy of gentamicin in five RDEB patients carrying nonsense mutations [37]. Gentamicin was administered either topically as a 0.1% ointment applied three times daily for two weeks to open skin lesions or intradermally as an 8 mg injection into intact skin over two days. Both routes of administration induced re-expression of C7 and the formation of anchoring fibrils (AFs) at the DEJ, as confirmed by immunofluorescence and electron microscopy. The restored C7 persisted for up to three months, reaching levels between 20% and 165% of that seen in normal skin. Clinically, topical gentamicin improved wound healing, enhanced dermal–epidermal adhesion, and reduced blister formation, with most treated lesions showing 85–100% closure at three months. Importantly, the treatment was well-tolerated, with no adverse effects or induction of anti-C7 autoantibodies observed.

In addition to our study, two more clinical studies have since investigated the effects of topical gentamicin in patients with RDEB. In a study by Mahajan et al., twelve patients received 0.1% gentamicin cream in a collagen base applied twice daily to one side of the body, with the opposite side left untreated as a control to compare wound healing progression [38]. Eight patients also completed pre- and post-treatment biopsies of the treated wounds, with mean fluorescence intensity (MFI) of C7 measured in both instances. After 12 weeks of treatment, there was a notable 173.69% increase in MFI in the three patients who harbored nonsense mutations, compared to 48.77% in patients without such mutations. While results did not reach statistical significance—likely due to the small sample size—the findings support the potential for topical gentamicin treatment. Similarly, Sandanger et al. conducted a phase 1/2 clinical trial in which four RDEB patients with nonsense mutations received 0.1% topical gentamicin ointment once daily for six weeks on all wounds on one body half, with the other half serving as a control [39]. While no significant improvements in wound healing were observed, immunohistochemistry confirmed successful PTC suppression in two patients, suggesting that gentamicin-induced readthrough may still occur at the molecular level even in the absence of a measurable clinical response.

Our research group conducted another phase 1/2 clinical trial investigating the effects of IV gentamicin in three RDEB patients with nonsense mutations in *COL7A1* and reduced baseline expression of C7 at the DEJ [40]. Participants received gentamicin at a dose of 7.5 mg/kg daily for 14 days, with two of the three patients continuing on a twice-weekly regimen for an additional 12 weeks. Post-treatment skin biopsies showed increased C7 expression at the DEJ in all participants, with newly synthesized collagen persisting for at least six months. Clinically, wound healing improved, with over 85% closure observed in all monitored wounds at one- and three-months post-treatment. Additionally, all patients demonstrated decreased disease severity, as reflected by reductions in Epidermolysis Bullosa Disease Activity and Scarring Index (EBDASI) total activity scores. The treatment was well-tolerated, with no adverse events or development of anti-C7 autoantibodies reported.

### 3.3. Studies on JEB

In preclinical studies studying JEB, gentamicin and paromomycin have successfully induced readthrough of *LAMB3* or *COL17A1* nonsense mutations, restoring laminin β3 or type XVII collagen (C17) production, respectively. For gentamicin, we assessed the potential of gentamicin to promote PTC readthrough in Herlitz junctional epidermolysis bullosa (H-JEB) keratinocytes lacking laminin β3, which were transfected with expression vectors carrying eight different *LAMB3* nonsense mutations [41]. Our results showed that gentamicin induced PTC readthrough across all eight tested mutations. After using lentiviral vectors to create stable H-JEB cell lines harboring the p.Arg635* and p.Cys290* nonsense mutations, gentamicin treatment led to a dose-dependent and sustained production and secretion of full-length laminin β3. Notably, the gentamicin-induced laminin β3 facilitated the reassembly, secretion, and deposition of laminin 332 at the DEJ. Furthermore, the restored laminin 332 corrected the defective cellular phenotype of H-JEB cells, improving cell morphology, growth potential, substrate adhesion, and reducing hypermotility. For paromomycin, Has et al. evaluated paromomycin in keratinocytes derived from patients with JEB carrying four different homozygous *COL17A1* nonsense mutations that lacked detectable C17 expression [28]. The response to paromomycin, gentamicin, and G418 varied by the sequence of the PTC and the nucleotide base immediately downstream of the stop codon. To optimize therapeutic outcomes, the same group developed a readthrough cocktail (TRID-C5), which included low doses of gentamicin and paromomycin along with apocynin, melatonin, CC-90009, and NMDI-14 [29]. TRID-C5 restored approximately 20% of C17 expression in JEB keratinocytes compared to normal cells and significantly improved cell adhesion, without inducing toxicity or impairing in vitro wound closure. Importantly, this cocktail also upregulated *COL17A1* mRNA, supporting both transcriptional and translational rescue.

Clinical studies of JEB have chiefly examined gentamicin; exploring topical, intramuscular (IM), or IV formulations. In our phase 1/2 clinical trial, three JEB patients with at least one nonsense mutation in *LAMA3* or *LAMB3* received 0.5% topical gentamicin applied twice daily for two weeks to lesional skin [42]. Topical gentamicin led to increased expression of laminin-332 chains—ranging from 40 to 93% for α3, 49 to 120% for β3, and 55 to 102% for γ2—at the DEJ, with expression sustained for at least three months. Clinical improvement was observed in the form of significant wound closure, with at least 85% healing in most treated areas at one- and three-months post-treatment. Importantly, the treatment was well-tolerated, with no adverse effects and no development of anti–laminin-332 autoantibodies detected in the blood or skin. Two additional case reports also demonstrated successful use of topical gentamicin in the treatment of JEB. In one case, a 6-month-old male JEB patient carrying a nonsense mutation of *COL17A1* applied 0.3% gentamicin ointment once daily to the left hand, arm, leg, and foot for 90 days, while a placebo ointment base was applied to the trunk [43]. The treated areas showed improved wound closure, reduced blister formation, and a significant reduction in the Birmingham Epidermolysis Bullosa Severity score (from 12.13 to 6.38), with no relapse for at least two months after treatment cessation. Immunohistochemical staining confirmed re-expression of C17 at the treated sites. In the second case, a 26-year-old female JEB patient carrying a nonsense mutation in *LAMB3*, with chronic ocular complications from JEB, applied gentamicin ointment once daily to the eyes in the evening for one week [44]. Prior to treatment, immunofluorescence of conjunctival cells showed markedly reduced laminin-332 expression. Post-treatment, there was significant improvement in corneal erosions, a decrease in fluorescein-stained punctate erosions, and a marked increase in laminin-332 expression—up to three to four times that of normal controls. These cases provide further clinical evidence for topical gentamicin as a potential therapeutic option in JEB.

For systemic administrations of gentamicin to treat JEB, Hammersen et al. conducted a retrospective review of systemic gentamicin treatment (7.5 mg/kg daily, administered intravenously or intramuscularly for 21 days) in five infants with severe-JEB caused by p.Arg635* hotspot mutations in *LAMB3* [45]. Treatment was well tolerated—serum gentamicin and creatinine stayed within safe ranges, and no extended hospital stays were required. However, post-treatment biopsies detected only trace laminin-332 (about 5% of normal) in a single patient, and treatment was unable to reverse the lethal course of disease. Despite this, parents still noted less skin fragility and improved quality of life in four of the five patients. Our research group further investigated the efficacy and safety of IV gentamicin in our open-label phase 1/2 clinical trial involving five pediatric JEB patients harboring nonsense mutations in *LAMA3* or *LAMB3* and decreased baseline expression of laminin-332 [46]. Our trial assessed two dosing regimens: three patients received 7.5 mg/kg/day for 14 days, and two received 10 mg/kg/day for 24 days. Immunofluorescence one month post-treatment confirmed increased expression of laminin-332 at the DEJ in all five patients. In the low-dose group, expression of the α3, β3, and γ2 chains increased by 30.0–58.3%, 46.7–77.0%, and 31.5–52.4%, respectively, while in the high-dose group, increases ranged from 57.5–100% for α3, 73.0–87.9% for β3, and 56.7–98.5% for γ2. Clinically, wound healing improved significantly: by three months, eight of nine wounds in the low-dose group and all wounds in the high-dose group achieved over 85% closure. All three patients assessed using EBDASI showed clinically meaningful reductions in disease activity. The treatment was well-tolerated, with no reported ototoxicity, nephrotoxicity, or development of anti–laminin-332 autoantibodies.

### 3.4. Studies on EB Simplex

In addition to the aforementioned clinical studies demonstrating efficacy of gentamicin in RDEB and JEB, a case report has also shown gentamicin’s potential in treating EB simplex with muscular dystrophy [47]. A female patient in her 30s with a *PLEC1* nonsense mutation received two cycles of IV gentamicin at 7.5 mg/kg/day for two weeks. Before treatment, she experienced mucocutaneous involvement, skeletal and respiratory muscle weakness, and significant myalgia. Following treatment, immunofluorescence staining of skin biopsies showed restored plectin expression at the DEJ, increasing from undetectable levels to 30% after the first cycle and 60% after the second. Plectin remained detectable for at least five months post-treatment. Although improvements in skeletal and respiratory muscle function were minimal, the patient experienced substantial relief from myalgia and improved quality of life, with reductions in pain and anxiety scores on the European Quality of Life 5-Dimensions 3-Level Version scale. The treatment was well-tolerated, with no nephrotoxic or ototoxic side effects observed.

### 3.5. Limitations and Future Directions with Aminoglycoside Therapy

Although gentamicin has demonstrated effective readthrough in various genetic disease models, including EB, its long-term use is limited by well-documented side effects such as ototoxicity, nephrotoxicity, and, to a lesser extent, retinal toxicity [48,49]. These toxicities, primarily driven by the generation of reactive oxygen and nitrogen species in sensitive tissues like the inner ear, can lead to cellular damage and death. Due to these risks, increasing efforts have focused on identifying alternative therapies, or identifying adjunct therapies, to enhance gentamicin’s readthrough efficacy while minimizing its toxic effects, which will be discussed in the next sections.

## 4. Evaluating Novel Readthrough Agents for EB in Preclinical Studies

### 4.1. Aminoglycoside Derivatives

One promising readthrough agent under investigation is ELX-02, a synthetic, non-antibiotic aminoglycoside analogue engineered to enhance PTC readthrough while minimizing the toxicity commonly associated with traditional aminoglycosides [50]. ELX-02 selectively targets eukaryotic ribosomes, reducing off-target effects on mitochondrial translation—a key contributor to aminoglycoside-induced toxicity [50,51]. Preclinical studies have shown that ELX-02 exhibits a favorable safety profile with fewer adverse effects than its aminoglycoside counterparts [52,53,54]. Our group recently demonstrated that ELX-02 induced a dose-dependent increase in the production of C7 and laminin β3, exceeding the levels observed with gentamicin treatment, in primary keratinocytes and fibroblasts derived from patients with RDEB- and JEB-harboring nonsense mutations [55]. It also reversed the abnormal hypermotility seen in RDEB and JEB cells and improved the deficient cell-substratum adhesion characteristic of JEB cells. Notably, the C7 and laminin 332 produced in response to ELX-02 localized correctly to the DEJ. This study is the first to show that ELX-02 can PTC readthrough and restore functional C7 and laminin 332 in RDEB and JEB models caused by nonsense mutations. Although ELX-02 has not yet been clinically evaluated in EB, it is currently in phase 2 trials for nephropathic cystinosis and Alport syndrome, highlighting its potential as a safer alternative readthrough therapy [52,56,57].

### 4.2. Ataluran/PTC124

Ataluren (also known as PTC124 or Translarna™) is a synthetic small molecule identified in 2007 via an ultra-high-throughput screen of over 800,000 compounds using a firefly luciferase reporter gene [58]. Structurally distinct from aminoglycosides, it was initially selected for its ability to promote readthrough of PTCs while displaying low toxicity and favorable pharmacological properties. Unlike aminoglycosides, ataluren’s mechanism of action involves inhibiting the eRF3/eRF1a release factor complex, preventing premature termination [59,60].

Ataluren has been investigated in a variety of preclinical models targeting nonsense mutations. In DMD, it demonstrated dose-dependent readthrough in primary muscle cells and dystrophin-deficient zebrafish, with efficacy varying by stop codon type [58,61]. In CF, outcomes were mixed: some studies reported CFTR restoration in organoids and intestinal glands, while others, including forskolin-induced swelling assays in p.Gly542* mouse organoids, showed no benefit [26,62]. Ataluren has successfully induced readthrough in other genetic diseases such as Usher syndrome, Shwachman–Diamond syndrome, retinitis pigmentosa, and neuronal ceroid lipofuscinoses [63,64,65,66]. However, other investigations in cell models for obesity, peroxisome biogenesis disorders, long-QT syndrome, and CF revealed little to no readthrough, and conflicting results often stemmed from differences in assay systems, mRNA structure, and cellular context [67,68,69]. Furthermore, subsequent studies suggested that ataluren’s initial identification may have been confounded by its stabilizing interaction with luciferase itself, leading to false-positive results [70,71]. Overall, while ataluren’s efficacy varies widely, its preclinical profile underscores its context-dependent activity and limitations.

Ataluren’s clinical development has been most extensively pursued in DMD and CF. In DMD, early-phase trials demonstrated improved dystrophin levels and delayed disease progression, leading to conditional approval by the European Medicines Agency (EMA) in 2014 for ambulatory pediatric patients ≥ 2 years [72]. However, this conditional approval was not renewed, and the EMA formally withdrew this authorization in March 2025. In CF, multiple large randomized controlled trials failed to show significant improvements in lung function or reduction in exacerbations [73]. Concomitant use of tobramycin in some studies raised concerns about antagonistic interactions, prompting a subsequent trial excluding aminoglycosides, which also showed no benefit [74]. Conflicting outcomes from these trials—along with two studies in which ataluren combined with ivacaftor failed to demonstrate clinical benefit—led to the suspension of ataluren for treating CF [75]. Currently, there is an ongoing trial with ataluren in combination with pembrolizumab for treating mismatch repair-deficient cancers, aiming to promote neoantigen generation via PTC readthrough (NCT04014530). Despite the mixed outcomes, ataluren’s approval in DMD remains a milestone, as it represents the only readthrough-inducing therapy to have ever received regulatory approval for clinical use.

In the context of EB, ataluren has shown mixed results in preclinical and clinical studies. In our unpublished data, we tested ataluren on our RDEB keratinocytes and fibroblasts carrying five distinct nonsense mutations. However, we did not observe any readthrough activity across a wide range of concentrations. Our findings align with a study by McElroy et al. demonstrating that ataluren failed to induce readthrough in two C7 cDNA constructs containing nonsense mutations [71]. In the context of JEB, in the aforementioned study by Has et al. evaluating the impact of various readthrough therapies on JEB keratinocytes harboring *COL17A1* nonsense mutations, in addition to paromomycin, ataluren was among the evaluated compounds [28]. While aminoglycosides yielded variable levels of C17 expression, ataluren did not restore protein expression in any tested cell lines. However, despite limited preclinical efficacy, ataluren has been successfully used clinically in a single case of JEB. An 11-year-old male JEB patient with a nonsense mutation in *LAMB3* was treated with oral ataluren two to three times daily over a 46-month period [76]. The therapy was well tolerated and associated with reduced frequency and duration of hospitalizations. Immunofluorescence revealed the reappearance of low levels of laminin-332, suggesting partial therapeutic effect, although quantitative protein assessment was limited by the patient’s decision to decline further biopsies. This case represents the first reported use of ataluren in EB patients and supports the possibility of clinical benefit in select patients, albeit with caution given the modest molecular response and absence of confirmatory protein quantification. Currently, the same study investigators have obtained an FDA-investigational new drug approval for ataluren treatment in three EB patients: 1 with EB Simplex with muscular dystrophy, 1 with JEB, and 1 with RDEB, with clinical outcomes pending.

### 4.3. Amlexanox

Inhibition of NMD has emerged as a potential therapeutic strategy for genetic disorders caused by PTCs, particularly when truncated proteins retain partial or full function. By stabilizing PTC-containing transcripts, NMD inhibitors can increase the production of protein from mutant genes [77]. One such inhibitor is amlexanox, an FDA-approved drug initially used to treat recurrent aphthous ulcers [78]. Amlexanox has demonstrated promising effects across several preclinical disease models. In cell lines derived from patients with cancer, DMD, and CF, treatment with 25 μM of amlexanox stabilized PTC-containing mRNA and, in some cases, led to the production of full-length proteins such as dystrophin, p53, and CFTR [79]. In a Charcot–Marie–Tooth disease model, amlexanox restored *GDAP1* mRNA levels and protein expression in hiPSC-derived neuronal cells harboring UGA nonsense mutations [80].

In the context of EB, amlexanox has shown variable efficacy. In an in vitro model of RDEB, amlexanox promoted successful stop codon readthrough, restoring full-length C7 collagen expression in patient-derived keratinocytes [81]. In three-dimensional organotypic skin cultures, this translates to increased deposition of C7 at the DEJ. However, in the aforementioned study by Has et al. utilizing JEB keratinocytes with *COL17A1* nonsense mutations, the investigators found amlexanox did not produce detectable levels of full-length C17 by immunoblot, highlighting the context-specific limitations of this approach [28].

### 4.4. Translation Termination Factor Inhibitors

Translation termination in eukaryotic cells is facilitated by eukaryotic release factors eRF1 and eRF3a/b, which recognize stop codons, mediate release of the nascent polypeptide, and promote ribosomal recycling [82,83]. These two factors also participate in the SURF (SMG1-UPF1-eRF1-eRF3) complex assembly involved in NMD. Recent therapeutic strategies have focused on targeting these termination factors to promote PTC readthrough. By reducing eukaryotic release factor levels through proteasome-mediated degradation, this induces ribosomal stalling at PTCs and favors translational readthrough [83,84].

CC-90009, a selective eRF3a degrader currently undergoing phase 1 trials for acute myeloid leukemia, has shown potent PTC readthrough activity in preclinical models of several genetic disorders, including mucopolysaccharidosis type I, DMD, CF, and retinoblastoma [83,85,86]. Notably, it synergizes with aminoglycosides and other novel compounds to significantly enhance readthrough efficacy [29,83,85,87]. Various eRF1 degraders have also demonstrated effectiveness in models of CF, hemophilia, and Hurler syndrome [84,88,89]. One eRF1 degrader, SRI-41315, modestly increased CFTR expression on its own, but markedly improved both protein expression and function when combined with G418, including in primary bronchial epithelial cells from CF patients [89].

In EB, both CC-90009 and SRI-41315 have shown promise in restoring protein function through PTC suppression in preclinical studies. Two studies demonstrated effective PTC readthrough in JEB keratinocytes harboring *COL17A1* nonsense mutations using CC-90009, either with G418 or as part of a readthrough cocktail that included aminoglycosides and NMD inhibitors [29,85]. In our own investigations, a combination therapy using low-dose gentamicin with either CC-90009 or SRI-41315 restored full-length C7 in RDEB keratinocytes and fibroblasts, and laminin 332 in JEB keratinocytes [90,91]. This treatment also corrected abnormal cellular phenotypes associated with RDEB or JEB such as hypermotility and poor substratum adhesion. Importantly, the restored proteins localized appropriately to the DEJ in three-dimensional organotypic skin equivalents. These findings support the potential of CC-90009 and SRI-41315, especially in combination with low-dose gentamicin, as promising therapeutic options for inherited skin disorders caused by nonsense mutations.

## 5. Other Promising Readthrough Therapies

Among emerging readthrough therapies, tRNA post-transcriptional inhibitors such as 2,6-diaminopurine (DAP) have shown notable promise. Mechanistically, when a ribosome reaches a PTC, tRNAs undergo critical post-transcription modifications to ensure accurate codon recognition. Among the enzymes modifying tRNAs, FtsJ RNA 2′-O-methyltransferase (FTSJ1) exclusively modifies tRNAs involved in stop codon UGA readthrough. DAP, originally identified through a system using a PTC-carrying firefly luciferase mRNA, targets FTSJ1 to inhibit UGA stop codon recognition [92]. By disrupting this modification, DAP enhances translational readthrough past UGA-associated PTCs. DAP demonstrated greater efficiency than G418 in restoring p53 function in a cancer cell line with an endogenous UGA nonsense mutation in the *TP53* gene [92]. In vivo, DAP reduced tumor growth in mice carrying *TP53*-UGA mutations and showed efficacy in CF models using patient-derived organoids and cells [93]. In the case of EB, our recent studies revealed that DAP significantly increased the synthesis of C7 in RDEB cells harboring UGA nonsense mutations in a dose-dependent manner, achieving superior efficacy compared to high-dose gentamicin [94]. Additionally, the restored C7 reversed the abnormal hypermotility characteristic of RDEB cells, indicating functional recovery [94]. DAP’s favorable pharmacokinetic profile—including high bioavailability in lung, brain, and muscle—makes it an attractive candidate for further development.

NV848 is a synthetic derivative of ataluren and shares a similar mechanism of action with DAP [95]. These compounds have shown readthrough activity across several CF model systems and promote tryptophan (Trp) incorporation at UGA PTCs [96]. Preclinical data indicate favorable safety profiles, with high-dose NV848 administration well tolerated in mouse models [96]. In vivo, NV848 improved CFTR protein expression and localization in CFTR homozygous p.Gly5* mice, with immunohistochemistry confirming restored expression in pulmonary duct epithelial cells [97]. Additionally, NV848 was able to restore Shwachman–Bodian–Diamond syndrome protein expression in Shwachman–Diamond syndrome-derived stem cells, showing broad applicability [64]. These results support NV848 as a promising candidate for nonsense mutation-related diseases, though they have not yet been evaluated in EB models.

SJ6986 is a cereblon E3 ubiquitin ligase modulator that promotes proteasome-mediated degradation of eRF3a, much like CC-90009 [83]. In CFTR-deficient epithelial cells harboring PTCs, SJ6986 restored CFTR function to approximately 20% of wild-type levels when used alone, and up to 50% when combined with G418 [83]. Despite these promising results, SJ6986 in CF models, and similarities in mechanism of action with another successful readthrough drug CC-90009, it has yet to be explored in the context of EB.

Clitocine is a nucleoside analog derived from mushrooms that substitutes for adenosine during transcription, promoting suppression of nonsense mutations [98]. In *TP53*-mutated cancer models, clitocine restored full-length p53 protein and significantly suppressed tumor growth in vivo. In unpublished data, clitocine demonstrated superior efficacy to G418 in suppressing the *LAMB3* p.Arg635* hotspot mutation. Specifically, clitocine at 1 μM induced a three-fold higher luciferase signal than G418 in a reporter system [99].

A recent study conducted a comprehensive comparison of readthrough efficacy across 5,837 PTCs using a high-throughput reporter library, comparing readthrough drugs CC-90009, SRI-41315, DAP, SJ6986, clitocine, gentamicin, G418, and 5-fluorouridine [100]. DAP, SJ6986, and clitocine emerged as the most broadly effective compounds, achieving > 2% readthrough in 31.4%, 21.3%, and 11.7% of PTCs, respectively—outperforming G418. Notably, each compound exhibited preferential activity based on stop codon type and gene context: DAP was most effective for UGA-rich genes (e.g., *ATRX*, *FAT1*), clitocine excelled in UAA-rich genes (e.g., *APC*, *BRCA2*), and SJ6986 was optimal for UAG-rich genes (e.g., *TSC2*, *MYBPC3*). In *TP53*, SJ6986 was the most effective compound for 36 of 102 nonsense mutations, followed by DAP (25) and clitocine (21). The findings emphasize the importance of tailoring readthrough therapy to the specific PTC, and position DAP, SJ6986, and clitocine as promising candidates for further testing in EB models [100].

## 6. Limitations in Amino Acid Selection at PTC Sites During Readthrough Induced by Small Molecules

The suppression of PTCs is mediated by a mismatch between the stop codon and a near-cognate aminoacyl-tRNA. While PTC readthrough is essential to restore full-length protein synthesis, the functionality of the resulting protein also depends on which amino acid is incorporated at the site of the PTC. Based on the standard genetic code, only a limited subset of natural suppressor tRNAs (near-cognate tRNAs) can theoretically mediate this readthrough. However, how small-molecule drugs influence the selection of these near-cognate tRNAs remains unclear.

At the third (wobble) codon position, certain readthrough therapies facilitate selective readthrough of specific stop codons. For the UGA stop codon, readthrough therapies most frequently promote the insertion of Trp, followed by arginine and cysteine. In the case of UAG, glutamine (Gln) is most commonly inserted, followed by tyrosine (Tyr) and lysine (Lys). For UAA, readthrough most often results in the incorporation of Gln, then Tyr, and less frequently Lys.

Readthrough-mediated amino acid insertion at a PTC can yield either a fully functional native protein or a variant differing by a single amino acid. When the resulting protein is altered, additional studies are required to assess its functionality and stability.

Given the mechanism of PTC readthrough, there is a possibility that the resulting protein may have altered function due to incorrect amino acid incorporation. Indeed, one study found that depending on phenotype severity, 5–10% of RDEB patients had missense mutations in the *COL7A1* gene caused by the substitution of a single amino acid [101].

In our studies involving RDEB and JEB models, we demonstrated that proteins produced through gentamicin- or ELX-02–induced readthrough of nonsense mutations retain functional activity in vitro [27,41,55]. Treatment with gentamicin or ELX-02 corrected hypermotility in both RDEB fibroblasts and JEB keratinocytes, indicating that restored expression of C7 and laminin 332 supports recovery of specific fibroblast and keratinocyte functions, thereby correcting the abnormal cellular phenotypes associated with RDEB and JEB. Additionally, gentamicin or ELX-02 stimulated laminin 332 production, successfully rescuing defective cell-substratum adhesion in JEB cells. Proper localization of C7 and laminin 332 to the DEJ in RDEB and JEB skin equivalents, respectively, further supports that gentamicin or ELX-02 induces correctly targeted, functional proteins.

In our in vivo studies with RDEB patients, it is essential to determine whether gentamicin-induced C7 expression results in clinical improvement. Our findings provide evidence of its functionality: the induced C7 formed normal AF structures in patients. Moreover, topical gentamicin, compared to placebo, enhanced dermal–epidermal adherence, promoted durable closure of erosive skin wounds, and reduced the formation of new blisters at treated sites [37,40]. Similarly, we also demonstrated that gentamicin-induced laminin 332 improved wound closure in JEB patients receiving IV or topical gentamicin [42,46].

## 7. Conclusions

Over the past two decades, the therapeutic landscape for nonsense mutation–driven EB has shifted from proof-of-concept reports with traditional aminoglycosides to a diverse portfolio of small-molecule, RNA-targeted, and protein-degradation strategies. Early clinical studies with gentamicin have already shown that restored expression of C7 or laminin-332 can translate into measurable wound closure and improved quality-of-life scores in selected patients. Parallel advances in aminoglycosides or its analog (e.g., ELX-02, paromomycin), translation termination factor degraders (CC-90009, SRI-41315, SJ6986), ataluren, tRNA post-transcriptional inhibitors (DAP, NV848), and nucleoside analogs (clitocine) now provide a mechanistically rich pipeline poised to address limitations of potency, codon specificity, and toxicity that have hampered first-generation compounds.

Readthrough therapy appears markedly more effective in EB than in other genetic disorders such as CF or DMD, largely because the target proteins in EB are exceptionally large, long-lived components of the basement membrane. For example, C7 assembles into 900-kDa trimers that form durable AFs, while laminin 332, likewise, integrates into stable extracellular scaffolds, with both proteins exhibiting slow physiological turnover. In our clinical trials, a short course of gentamicin restored C7 to over 50% of normal levels [37,40], and laminin 332 to over 40% of normal levels [42,46], with each persisting for at least three months—far longer than the transient gains typically seen for CFTR or dystrophin. This intrinsic protein stability suggests that intermittent, short-pulse gentamicin could maintain functionally adequate levels of C7 in RDEB and laminin 332 in JEB, making EB uniquely suited to sustain benefit from readthrough therapy.

Looking ahead, reduced toxicity and enhanced readthrough efficiency will be critical to advancing the long-term safety and effectiveness of readthrough therapies. Structural optimization of existing compounds and the rational design of new agents represent promising strategies toward this goal. However, a major challenge remains: the individual threshold of restored protein required to achieve durable clinical improvement is highly variable across different diseases, tissues, and patients—and is often unknown. For severe genetic conditions with high disease burdens, readthrough therapy alone may not fully reverse disease manifestations but offers meaningful potential as part of a combination approach. In the context of EB, where both cutaneous and life-threatening extracutaneous complications exist, readthrough therapy holds particular promise as a systemic treatment strategy capable of addressing multiple disease domains simultaneously. Continued development of efficient, safe, and scalable readthrough therapies, therefore, represents a critical frontier for future research efforts in EB.

## Data Availability

Not applicable.

## Abbreviations

The following abbreviations are used in this manuscript:

DAP	2,6-diaminopurine
AFs	Anchoring fibrils
ATM	Ataxia Telangiectasia Mutated
CF	Cystic fibrosis
CFTR	Cystic fibrosis transmembrane conductance regulator
DEJ	Dermal–epidermal junction
DMD	Duchenne Muscular Dystrophy
DEB	Dystrophic Epidermolysis Bullosa
EB	Epidermolysis Bullosa
EBDASI	Epidermolysis Bullosa Disease Activity and Scarring Index
FTSJ1	FtsJ RNA 2′-O-methyltransferase
Gln	Glutamine
H-JEB	Herlitz Junctional Epidermolysis Bullosa
IM	Intramuscular
IV	Intravenous
JEB	Junctional Epidermolysis Bullosa
Lys	Lysine
MFI	Mean Fluorescence Intensity
NMD	Nonsense-Mediated Decay
PTC	Premature Termination Codons
RDEB	Recessive Dystrophic Epidermolysis Bullosa
Trp	Tryptophan
C7	Type VII collagen
C17	Type XVII collagen
Tyr	Tyrosine
